# Lycopene Protects against Smoking-Induced Lung Cancer by Inducing Base Excision Repair

**DOI:** 10.3390/antiox9070643

**Published:** 2020-07-21

**Authors:** Junrui Cheng, Baxter Miller, Emilio Balbuena, Abdulkerim Eroglu

**Affiliations:** 1 Plants for Human Health Institute, North Carolina State University, 600 Laureate Way, Room 3204, Kannapolis, NC 28081, USA; jcheng26@ncsu.edu (J.C.); blmille4@ncsu.edu (B.M.); ejbalbue@ncsu.edu (E.B.); 2 Department of Molecular and Structural Biochemistry, College of Agriculture and Life Sciences, North Carolina State University, 351 Polk Hall, Raleigh, NC 27607, USA

**Keywords:** lycopene, β-carotene, lung cancer, base excision repair, gap junction communication, all-trans retinoic acid, oxidative stress

## Abstract

Background: Oxidative stress plays a critical role in lung cancer progression. Carotenoids are efficient antioxidants. The objective of this study was to explore the efficacy of all-trans retinoic acid (ATRA) and carotenoids in cigarette smoke-induced oxidative stress within A549 human lung cancer epithelial cells. Methods: A549 cells were pretreated with 1-nM, 10-nM, 100-nM, 1-μM and 10-μM ATRA, β-carotene (BC) and lycopene for 24 h, followed by exposure to cigarette smoke using a smoking chamber. Results: The OxyBlot analysis showed that smoking significantly increased oxidative stress, which was inhibited by lycopene at 1 nM and 10 nM (*p* < 0.05). In the cells exposed to smoke, lycopene increased 8-oxoguanine DNA glycosylase (OGG1) expression at 1 nM, 10 nM, 100 nM, and 1 μM (*p* < 0.05), but not at 10 μM. Lycopene at lower doses also improved Nei like DNA glycosylases (NEIL1, NEIL2, NEIL3), and connexin-43 (Cx43) protein levels (*p* < 0.05). Interestingly, lycopene at lower concentrations promoted OGG1 expression within the cells exposed to smoke to an even greater extent than the cells not exposed to smoke (*p* < 0.01). This may be attributed to the increased SR-B1 mRNA levels with cigarette smoke exposure (*p* < 0.05). Conclusions: Lycopene treatment at a lower dosage could inhibit smoke-induced oxidative stress and promote genome stability. These novel findings will shed light on the molecular mechanism of lycopene action against lung cancer.

## 1. Introduction

Lung cancer is the leading cause of cancer death, with eight million annual global deaths attributed to direct tobacco use, according to a report from World Health Organization (WHO) in 2019 [1]. Globally, 55% of lung cancer deaths in women and over 70% of lung cancer deaths in men are due to smoking [2]. Cigarette-smoking is one of the major environmental cues inducing the formation of reactive oxygen species (ROS) and the occurrence of oxidative stress, thereby resulting in damage to and modification of cellular macromolecules, and most importantly, genomic DNA that can lead to mutations [3]. However, to date, there are not yet effective treatments against smoke-induced oxidative stress or lung cancer.

Carotenoids are hydrophobic molecules synthesized mainly by plants and some microorganisms such as bacteria, algae or fungi [4,5]. β-carotene, α-carotene, β-cryptoxanthin, lycopene, lutein, and zeaxanthin are the major circulating carotenoids found in humans [6]. Two of the scavenger receptor class B (SRB) proteins, SR-B1 and CD36, have been implicated as carotenoid transporters in various tissues of animals [7,8]. The health-promoting effects of carotenoids were initially attributed to their antioxidant properties, at least in vitro, as they are efficient compounds in quenching free radical reactions [9]. Several studies highlighted the ability of carotenoids and their metabolites to regulate intracellular signaling cascades, therefore modulating gene expression and protein translation in metabolic pathways associated with inflammatory and oxidative stress [10,11]. Some preclinical and clinical studies have shown that carotenoids can prevent a wide variety of chronic diseases, including cancer [12]. Lycopene, its metabolites (i.e., apo-lycopenoids), and foods enriched with lycopene have shown protective effects against prostate cancer [13], hepatocellular carcinoma [14,15,16], and gastric cancer [17]. Lycopene is involved in the suppression of tumorigenesis via several pathways, including inhibiting the nuclear factor-κB signaling pathway, activating sirtuin 1 pathway or modulating the reverse cholesterol transport mechanism by inhibiting 3-hydroxy-3-methylglutaryl–coenzyme A reductase expression [18]. In addition, lycopene and its metabolites (i.e., acycloretinoic acid and apo-10′-lycopenoic acid) have been shown to activate retinoic acid receptor β (RARβ), leading to reduced cell proliferation [19,20,21], increased apoptosis [22], and enhanced gap junction communication (GJC) by upregulating connexin-43 (Cx43) [23]. Nevertheless, fewer studies have examined the effects of lycopene supplementation on lung cancer and the underlying molecular mechanisms [24].

8-Hydroxy-2-deoxyguanosine (8-OHdG) and 8-oxoguanine (8-oxoG) are the major oxidative modifications in DNA [25,26]. These 8-hydroxylated guanine species can be excised from the DNA by enzymatic repair systems [27], so both 8-OHdG and 8-oxoG can be measured as biomarkers of oxidative DNA damage [26,27,28]. Base excision repair (BER) is responsible for repairing the vast majority of endogenous DNA damage, including oxidations and single-strand breaks [29]. DNA glycosylases are within a family of enzymes involved in recognition of BER and excision of a damaged base. The 8-oxoguanine DNA glycosylase (OGG1) and Nth like DNA glycosylase 1 (NTH1) excise most the oxidatively damaged base lesions [30]. OGG1 recognizes and removes 8-OHdG to prevent further lesions to DNA [31,32]. In double-stranded DNA, one of the predominant substrates for OGG1 is 8-oxoG, while those for NTH1 are oxidized pyrimidines and formamidopyrimidines [29]. Nei like DNA glycosylases (NEIL) are distinct from NTH1 and OGG1 in structural features and reaction mechanisms. While most DNA glycosylases, including OGG1 and NTH1, are active only with duplex DNA, NEILs present a unique preference for excising lesions from a DNA bubble [30]. NEIL3, in comparison to NEIL1 and NEIL2, has weak glycosylase activity on duplex DNA and weak lyase activity on all substrates [29]. It is now clear that reduced activity of OGG1 is a risk factor in lung cancer. Moreover, the combination of smoking and low glycosylase activity was associated with a higher risk of lung cancer, which suggests a potential strategy for attenuating the smoke-induced lung cancer by targeting the BER-related genes [33].

We investigated the role of lycopene within smoke-induced oxidative stress in non-small cell lung cancer cells (A549 cells). We also compared the efficacy of lycopene in inhibition of lung oxidative stress along with β-carotene and ATRA, the biologically active form of vitamin A. We further explored the underlying molecular mechanism by examining BER-related genes.

## 2. Materials and Methods

### 2.1. Chemicals and Reagents

All-trans retinoic acid (R2625, purity ≥ 98%), β-carotene (C9750, purity ≥ 93%) and lycopene (75051, purity ≥ 85%) were purchased from Millipore Sigma (Burlington, MA, USA). Dulbecco’s modified Eagle’s medium (DMEM) with high glucose, L-glutamine (200 mM), penicillin–streptomycin (100X) and fetal bovine serum were all purchased from Thermo Fisher Scientific (Waltham, MA, USA). OxyBlot protein oxidation detection kit (Millipore Sigma); all the antibodies and luminol detection reagents (Santa Cruz Biotechnology—Dallas, TX, USA); PureLink RNA extraction kit (Thermo Fisher Scientific—Waltham, MA, USA) and Novo cDNA kit were purchased from BioVision (Milpitas, CA, USA). Applied Biosystems PowerUp SYBR Green Master Mix (preformulated, optimized universal 2× master mix for real time PCR workflows, radioimmunoprecipitation assay (RIPA) lysis and extraction buffer, cell lysis reagent for cultured mammalian cells, Halt Protease and Phosphatase Inhibitor Cocktail (100×) and Pierce Rapid Gold BCA protein assay kit were all obtained from Thermo Fisher Scientific (Waltham, MA, USA).

### 2.2. Cell Culture

**Figure 1:**
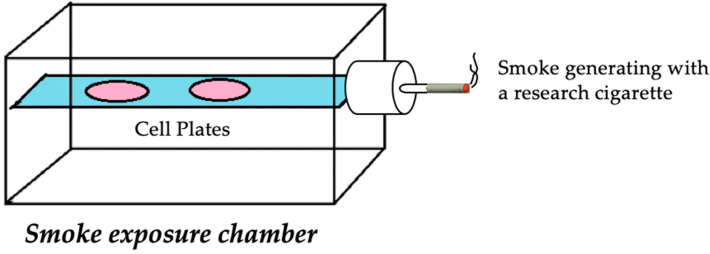
Schematic diagram of chamber apparatus for cigarette-smoke exposure. A549 cells seeded in 6-well plates (pink) with lids off were placed onto the platform (blue) inside a vacuumed-sealed plexiglass container. A fan inside the cylindrical portion propelled the smoke from the University of Kentucky research cigarette (filter removed) into the chamber to induce exposure.

A549 cells (human alveolar basal epithelial cells) were purchased from Millipore Sigma. The A549 cell line is commonly used as a model of lung carcinoma. Cancer cells were cultured in DMEM medium supplemented with 2 mM l-glutamine, 1% Pen–Strep, 10% FBS and maintained in a humidified incubator with 95% air and 5% CO_2_ at 37 °C. Cells were split at subconfluent cultures (80–90% confluent) 1:3 to 1:6, i.e., seeding density at 2–4 × 10^4^ cells/cm^2^ using trypsin-EDTA (% 0.25). For individual assays, cells were seeded into 6-well plates at a density of 2.5 × 10^5^ cells in 2 mL media in each well. ATRA and β-carotene were dissolved in ethanol, while dimethyl sulfoxide (DMSO) was used as a carrier solvent for lycopene; cells were pretreated with these compounds at different doses (1 nM, 10 nM, 100 nM, 1 μM, and 10 μM) for 24 h. Then, lids of the plates were removed to expose lung cancer cells to one cigarette smoke from University of Kentucky research cigarettes (brand 1A1, 25 mg tar, 0.3 mg nicotine, [34]) using the chamber shown in Figure 1. The time-course experiment (10, 20, 30, 40, 50 and 60 min) was carried out to determine the optimal duration of exposure, leading to selecting a time point of 50 min, which was published previously [35,36].

### 2.3. RNA Isolation

The PureLink RNA Mini Kit was used for RNA extraction from cultured A549 cells according to the manufacturer’s instructions. In brief: after aspirating off the medium, lysis buffer with 1% 2-mercaptoethanol was added to each well. All contents were then transferred to the RNase-free microcentrifuge tubes and mixed with 70% ethanol, followed by centrifugation at 2000× *g* for 2 min. Then, the supernatant was transferred to cartridges and spun down at 2000× *g* for 2 min. The flow-through was discarded. Two wash buffers were added to the cartridges at separate times, followed by spinning down at 2000× *g* for 15 s. Next, the cartridge was spun down at 12,000× *g* for 2 min to let RNA bind tightly to the cartridge and followed by addition of 50 μL DEPC water to the center of the cartridges. Finally, total RNA from each sample was collected after spinning the cartridges at 12,000× *g* for 2 min. RNA quality and quantity were assessed by the SpectraMax QuickDrop micro-volume spectrophotometer (Molecular Devices - San Jose, CA, USA).

### 2.4. cDNA Synthesis and Quantitative PCR

Novo cDNA kit was utilized to synthesize cDNA from 500 ng RNA using a Biometra TAdvanced 96G Thermal cycler system (Analytik Jena—Jena, Germany) program with conditions at 25 °C for 10 min, 42 °C for 15 min and 85 °C for 5 min. The newly synthesized first-strand cDNA was used as a template for assessing mRNA expression of target genes. Quantitative real-time RCR was carried out using a 20-μL reaction mixture; 10 μL 2X SYBR Green Supermix, 2 μL of 10-mM primer mix (including forward and reverse primers), 3 μL deionized water and 5 μL cDNA (diluted in RNase-free water). Cycling conditions were 50 °C for 2 min and 95 °C for 10 min; followed by 40 cycles at 95 °C for 15 s, 60 °C for 15 s, 95 °C for 1 min, then 55 °C and 95 °C for 30 s. Primers were designed using the Primer-BLAST tool at NCBI. Primer sequences are list in Supplementary Materials Table S1. RT-PCR was performed using SYBR quantification of gene expression, normalized to the levels of β-actin and then calculated by reference to the average values for the control group using the comparative Ct method. For each sample and each gene, PCR reactions were carried out in triplicate and repeated at least twice.

### 2.5. Western Blotting

Cell lysates were prepared by using RIPA buffer with protease inhibitors. Following cell lysis, proteins obtained from each sample were quantified using BCA Assay following manufacturer directions. Cell lysates (amounts equalized by protein concentration) were mixed with a 4 × NuPAGE LDS sample buffer and 10 × NuPAGE reducing agent (obtained from Thermo Fisher Scientific, Waltham, MA, USA) and then boiled for 7 min. Lysates were loaded on a 4–12% Bis-Tris gel with MOPS running buffer and electrophoresis was carried out according to standard protocols. Proteins were transferred to a nitrocellulose membrane by using iBlot 2 (dry transfer system) over the course of 7 min. Membranes were blocked at room temperature with 5% bovine serum albumin (BSA) *w/v* in Tris-buffered saline plus (TBS) supplemented with 0.05% Tween-20 (TBS-T) for 1 h. Then, membranes were incubated overnight in primary antibody conjugated with horseradish peroxidase (HRP) at 4 °C. The antibodies were diluted at different doses: OGG1 (1:3000), connexin-43 (1:2500), NEIL1 (1:5000), NEIL2 (1:2500), NEIL3 (1:2500), RARβ (1:1500) and β-actin (1:5000) in TBS-T with 5% BSA (*w/v*). After washing membranes in TBS-T (3 × 10 min), membranes were developed for visualization with a luminol enhancer solution. β-Actin was used as a loading control and evaluated using a UVP ChemStudio imaging system along with proteins of interest. All image analyses were performed using ImageJ software Version 1.8 (NIH, Bethesda, MD, USA).

### 2.6. OxyBlot

As a consequence of oxidative modification of proteins, carbonyl groups are introduced into protein side chains. The OxyBlot Kit from Sigma was employed to detect these carbonyl groups according to the manufacturer’s instructions. In short, 5-μL sample protein was denatured by 5 μL SDS and was then mixed with 10 μL 2,4-dinitrophenylhydrazine. After placing the mixture at room temperature for 20 min, a neutralization reagent was added to stop the reagent, followed by an addition of 2 μL loading buffer. The protein was then separated on a 4–12% Bis-Tris gel and transferred to nitrocellulose membranes. Nonspecific blocking was carried out by using 1% BSA for 1 h, followed by primary antibody blocking at 4 °C overnight. Finally, an HRP-conjugated secondary antibody was used for signal detection.

### 2.7. Statistical Analysis

Student’s *t*-test and analysis of variance (ANOVA) were performed to compare the mean of the control group to the mean of the treatment group. *p* < 0.05 was considered significant. Normality of distribution was examined by using D’Agostino–Pearson omnibus normality test. The data were considered as normally distributed if *p* > 0.05. Equality of variance was examined with an F test. *p* > 0.05 was considered as equal variances. The data shown are mean values of three independent experiments with error bars corresponding to standard errors. All statistical analysis was performed by using GraphPad Prism 8 (San Diego, CA, USA).

## 3. Results

### 3.1. Lycopene Inhibited Smoking-Induced Oxidative Stress

**Figure 2:**
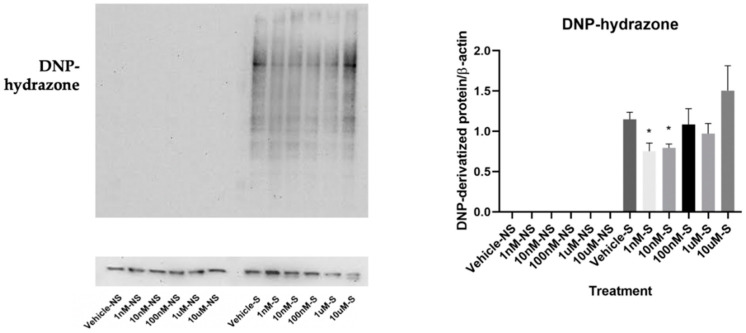
Effects of lycopene treatment at 1 nM, 10 nM, 100 nM, 1 μM, and 10 μM for 24 h on oxidative stress. Graphic representation of fold changes in the carbonyl groups that were introduced to protein side chains, quantified by using OxyBlot analysis. Three replicates were used for statistical analysis. Values are means ± SEMs. S—cells exposed to smoke; NS—cells not exposed to smoke; * indicates significance at *p* < 0.05 compared with cells exposed to smoke treated with vehicle.

By employing OxyBlot analysis, we found that the cells exposed to smoke presented a significantly higher level of DNP-hydrazone in comparison to the cells not exposed to smoke, which did not form protein carbonization at all, indicating more oxidized protein formation within the cells exposed to smoke. Interestingly, the number of carbonyl groups within the cells exposed to smoke decreased with lycopene treatment at 1 nM and 10 nM, but not at higher concentrations (Figure 2).

### 3.2. Lycopene Increased Base Excision Repair in the Cells Exposed to Smoke

**Figure 3:**
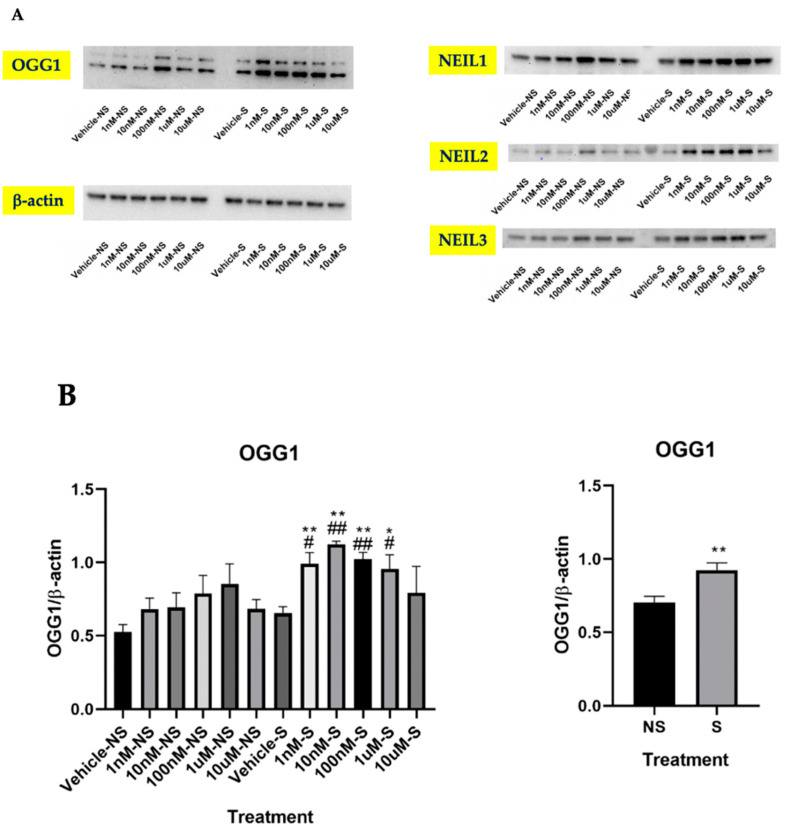

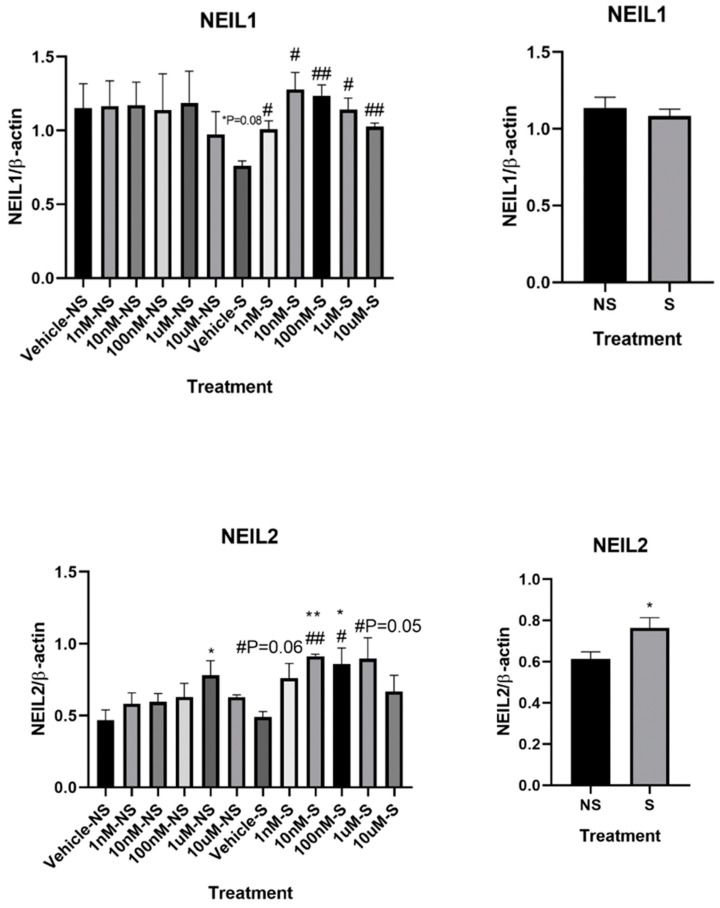

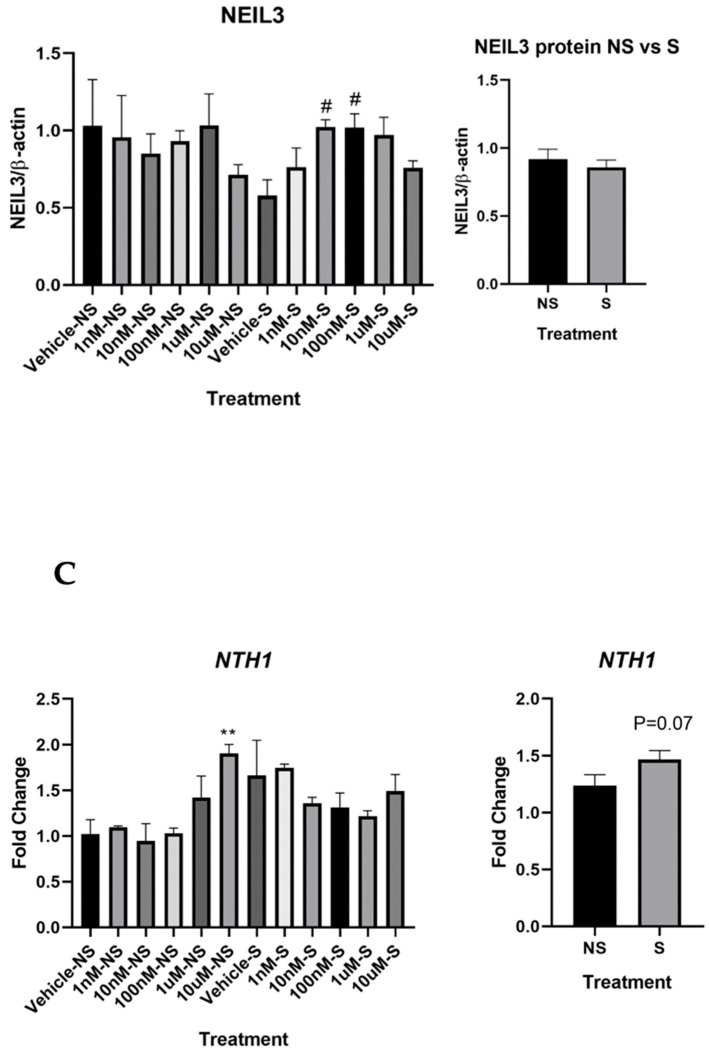
Effects of lycopene treatment at 1 nM, 10 nM, 100 nM, 1 μM, and 10 μM for 24 h on base excision repair-related genes. Graphic representation of fold changes in: (**A**,**B**) protein levels of 8-oxoguanine DNA glycosylase (OGG1), Nei-like glycosylase (NEIL)1, NEIL2 and NEIL3; and (**C**) mRNA level of NTH1. Three replicates were used for statistical analysis. Values are means ± SEMs. NS—cells not exposed to smoke; S—cells exposed to smoke; * indicates significance at *p* < 0.05; ** indicates significance at *p* < 0.01 compared with the cells not exposed to smoke treated with vehicle; # indicates significance at *p* < 0.05 compared with the cells exposed to smoke treated with vehicle.

By further exploring the underlying mechanism involving lycopene inhibition of smoke-induced oxidative stress, we found that OGG1 protein expression significantly increased in the cells treated with lycopene at 1 nM, 10 nM, 100 nM, and 1 μM, but not at 10 μM (Figure 3A,B). There was a trend of increasing OGG1 protein expression in the cells not exposed to smoke with lycopene treatment at 1 nM, 10 nM, 100 nM, and 1 μM, but the changes were not statistically significant. Consistently, our results showed that in the cells exposed to smoke, NEIL2 and NEIL3 protein concentrations were significantly enhanced with lycopene treatment at 10 nM and 100 nM. In the cells exposed to smoke, NEIL1 protein expression was elevated by lycopene treatment at all dosages, but the extent of such increase was more pronounced at 10 nM and 100 nM (Figure 3A,B). However, the mRNA expression of NTH1, another glycosylase involved in base excision repair, was not significantly changed in the cells exposed to smoke with lycopene treatment in comparison to the cells treated with vehicle (Figure 3C). Intriguingly, we found that at lower concentrations, lycopene treatment in the cells exposed to smoke promoted OGG1 expression to an even greater extent compared with the cells not exposed to smoke (Figure 3B). ATRA and BC treatment did not alter the expressions of OGG1, NEIL1, NEIL2 or NEIL3 (data not shown).

### 3.3. Lycopene Uptake Was Increased in the Cells Exposed to Smoke

**Figure 4:**
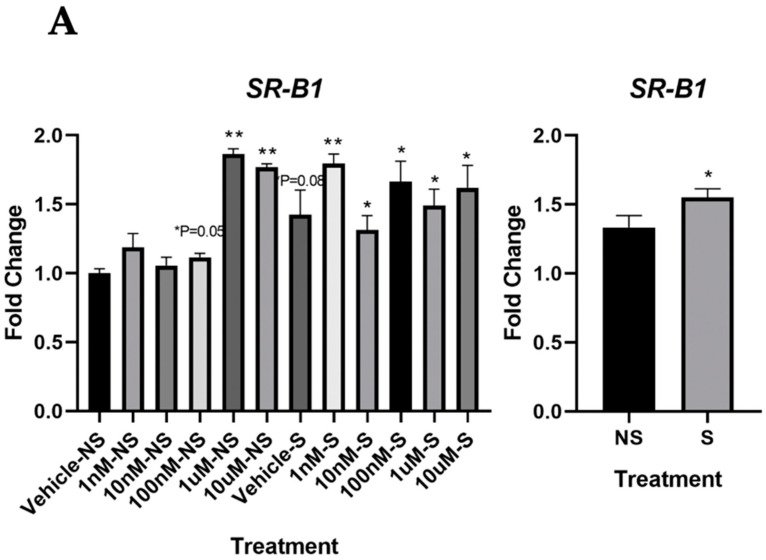

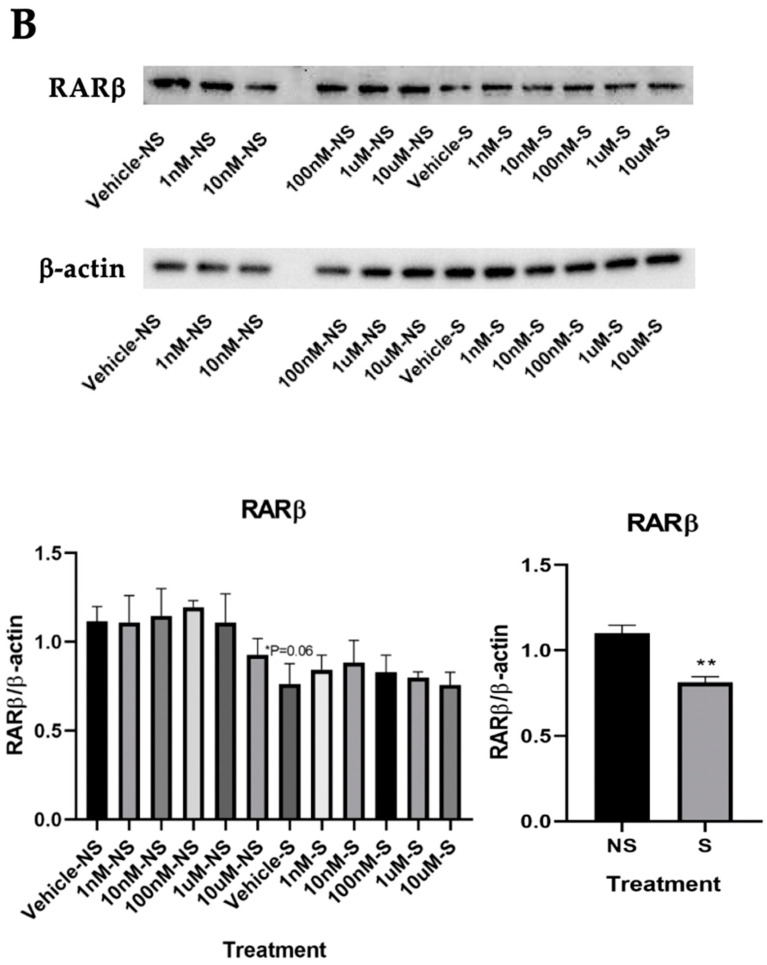
Effects of lycopene treatment at 1 nM, 10 nM, 100 nM, 1 μM and 10 μM for 24 h on carotenoid uptake and signaling pathway. Graphic representation of fold changes in: (**A**) mRNA level of SR-B1 and (**B**) protein levels of RARβ. Three replicates were used for statistical analysis. Values are means ± SEMs. NS—cells not exposed to smoke; S—cells exposed to smoke; cells treated with vehicle. Student’s *t* test was used to compare protein expressions between lycopene-treated cells and vehicle-treated cells; * indicates significance at *p* < 0.05 compared with the cells not exposed to smoke treated with vehicle; ** indicates significance at *p* < 0.01 compared with the cells not exposed to smoke treated with vehicle; # indicates significance at *p* < 0.05 compared with smoking. Two-way ANOVA was used to compare protein expressions between the cells exposed to smoke and the cells not exposed to smoke, * indicates significance at *p* < 0.05 compared with the cells exposed to smoke; ** indicates significance at *p* < 0.01 compared with the cells not exposed to smoke.

Since our data indicated that lycopene could initiate DNA repair more efficiently under oxidative stress, we proceeded to explore the underlying mechanism. Interestingly, we found that the mRNA level of SR-B1 was significantly increased in the smoking, compared with that in the cells not exposed to smoke, suggesting an increased carotenoid uptake in the cells under oxidative stress (Figure 4A). However, the mRNA expressions of cluster of differentiation (CD36), beta-carotene oxygenase 1 (BCO1), and beta-carotene oxygenase 2 (BCO2) were not changed with lycopene treatment (data not shown). In addition, we found that RARβ protein expression decreased in the Vehicle–S group, compared with that in the Vehicle–NS group (*p* = 0.06), but such a decrease in expression was attenuated with lycopene treatment in the cells exposed to smoke (Figure 4B).

### 3.4. Lycopene Increased Gap Junctional Communication

**Figure 5:**
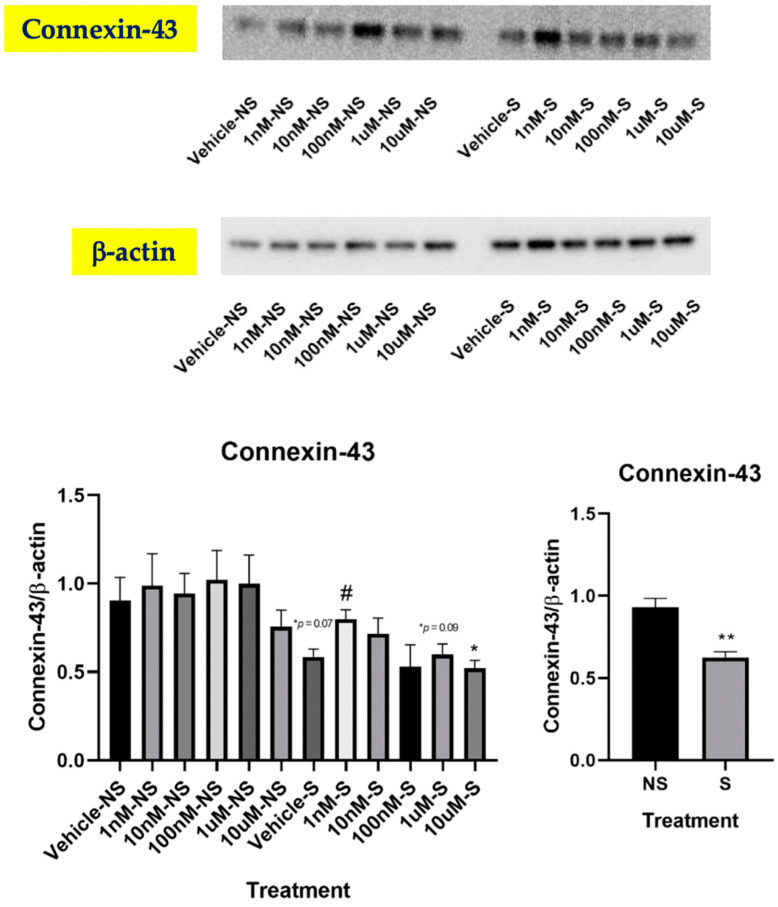
Effects of lycopene treatment at 1 nM, 10 nM, 100 nM, 1 μM and 10 μM for 24 h on Cx43. Graphic representation of fold changes in the protein expression of Cx43. Three replicates were used for statistical analysis. Values are means ± SEMs. NS—cells not exposed to smoke; S—cells exposed to smoke; cells treated with vehicle. Student’s *t* test was used to compare protein expressions between lycopene-treated cells and vehicle-treated cells; * indicates significance at *p* < 0.05 compared with the cells not exposed to smoke treated with vehicle; # indicates significance at *p* < 0.05 compared with smoking. Two-way ANOVA was used to compare protein expressions between the cells exposed to smoke and the cells not exposed to smoke; ** indicates significance at *p* < 0.01 compared with the cells not exposed to smoke.

Stimulation of gap junction communication (GJC) has been suggested to be one of the biochemical mechanisms underlying the cancer-preventive activity of carotenoids [37]. Previous studies have reported that lycopene could enhance GJC by upregulating connexin-43 (Cx43), which attenuated oral cancer [23] and prostate cancer [38,39] in in vitro settings. Therefore, we sought to investigate whether lycopene could decrease cigarette smoke-induced oxidative stress by promoting gap junction protein alpha-1 protein (also known as Cx43). Intriguingly, we found that the Cx43 protein level was increased in the cells exposed to smoke with lycopene treatment at 1 nM, but not at other dosages (Figure 5). Nevertheless, even with lycopene treatment, Cx43 protein expression was not restored in the cells exposed to smoke to the same level as non-smoking (*p* < 0.01), indicating that GJC induction may not be the main mechanism by which lycopene inhibited smoke-induced oxidative stress. ATRA and BC treatment did not change Cx43 expression in the current study (data not shown).

## 4. Discussion

Previous studies have shown that lycopene supplementation attenuated cell proliferation and growth, DNA damage, and insulin-like growth factor-1 stimulated growth [24]. However, the role of lycopene in smoke-induced oxidative stress was still unclear. To our knowledge, the present study provided the first evidence that lycopene inhibited cigarette smoke-induced lung oxidative stress by targeting BER-related genes.

Our OxyBlot analysis showed a marked increase in carbonyl groups introduced into protein side chains within the cells exposed to smoke, which verified our in vitro model of cigarette smoke exposure. Mounting evidence from in vitro and in vivo studies shows that lycopene and its metabolites have antioxidant and anti-inflammatory properties against carcinogen-induced lung lesions [15,40,41,42] by inhibiting lipid peroxidation, DNA damage, production of inflammatory cytokines (i.e., TNFα, IFNγ, IL-10) [22,23], and enhancing the activities of two major antioxidant enzymes, superoxide dismutase and catalase [41,42].

In the present study, we also reported beneficial effects of lycopene against cigarette smoke-mediated oxidative stress. The dosages of lycopene used in this study (1 nM–10 μM) were within physiological relevance [41]; and the protective effect of lycopene was only observed at 1 nM and 10 nM. Several in vivo and human studies have shown that exposure to high doses of carotenoids has a pro-oxidant effect [43]. Although we did not observe higher oxidative stress in the high-dosage groups, we found that lycopene only exerted antioxidative effects at low-dosage, while such beneficial effects were diminished at high-dosage.

We showed that lycopene supplementation at lower doses significantly increased OGG1 protein levels. Such finding was consistent with the results from a randomized clinical trial, showing that in healthy adults, a supplementation of purified lycopene at 30 mg/d for eight weeks significantly decreased 8-OHdG. Cross-sectional observation from another human study reported that serum lycopene concentrations were inversely correlated with the OGG1 expression in prostate biopsies [44]. A sequential 1-week supplementation study of spaghetti sauce (126 g/day), tomato juice (450 mL/day), or oleoresin capsules (2.5 g/day) as sources of lycopene did not alter the level of 8-OHdG contents of lymphocyte DNA [45]. However, these studies focused on the OGG1 level in prostate tissue and lymphocytes, respectively, and lycopene may have different antioxidative effects in a tissue-specific manner due to its tissue-specific distribution [46]. Thus, the association between lycopene supplementation and lung OGG1 expression in humans warrants further investigation. Cx43 is a transmembrane protein that is responsible in part for intercellular communication between adjacent epithelial cells via gap junctions. Intercellular GJC plays a critical role in tissue homeostasis and the regulation of cell growth and differentiation [47]. Cancer cells often display a loss of GJC due to the dysregulated expression of connexin genes at multiple levels [48]. Indeed, several Cx43 knockout mouse models have supported the notion that Cx43 has a tumor-suppressive function in the lung [47], and evidence has shown that tobacco exposure resulted in a lower expression of Cx43 [49]. Consistently, our study reported decreased protein levels of Cx43 in the cells exposed to smoke. Furthermore, this is the first study showing that lycopene treatment at low doses can upregulate pulmonary Cx43, which may provide new insights into the mechanism of lycopene action at the molecular level for chemoprevention of lung cancer.

It is well established that tobacco exposure can reduce the expression of multiple DNA repair genes [50]. However, interestingly, our ANOVA results showed increased protein levels of OGG1 and NEIL2 as well as enhanced transcriptional level of NTH1 in the cells exposed to smoke compared with the non-smoking ones. Scavenger receptor type B class I (SR-B1) protein is responsible for the facilitate diffusion for the uptake of carotenoids, including lycopene [8]. By further exploring the underlying mechanism, we found that the transcriptional level of SR-B1 was significantly enhanced in the cells exposed to smoke. It has been shown that upon cigarette smoke exposure, SR-B1 will go through subcellular redistribution and ubiquitination, leading to a reduced cellular SR-B1 expression [36]. Intriguingly, we did not observe such a decline within the cells exposed to smoke upon lycopene treatment, leading us to hypothesize that cells under oxidative stress may tend to intake more lycopene in order to attenuate the smoke-induced oxidation once with the antioxidative carotenoids. Further studies, especially in vivo studies, are needed to confirm the role of lycopene in regulating SR-B1 under oxidative stress.

Previous studies have shown that RARβ was significantly decreased in the animals with cigarette smoke exposure [51]. Consistently, our study found that the RARβ protein expression was significantly lower in the cells exposed to smoke, in comparison to the cells not exposed to smoke. In specific, we observed a substantially decreased RARβ protein level in the Vehicle–S group, in comparison to the Vehicle–NS group (*p* = 0.06). However, lycopene treatment diminished such decrease. It has been reported that in non-small cell lung cancer (NSCLC) patients, the aberrant expression of RARβ was associated with increased NSCLC severity [52]. Recently, Izhar and colleagues showed that nine nuclear receptors, including RARβ, were localized to the sites of DNA damage after laser micro irradiation (a source of oxidative stress) [53]. Therefore, it is possible that lycopene induced DNA repair to prevent pulmonary carcinogenesis by enhancing RARβ expression. We are going to test this hypothesis as our future direction.

One major limitation of this study is that we did not directly examine DNA repair. Although analyzing the expressions of DNA repair enzymes as well as performing the OxyBlot study showed how BER was altered within the cells upon cigarette-smoke exposure and lycopene treatment, we admit that extra assays (i.e., comet assay and direct ROS quencher assay [54]) are warranted to further validate the antioxidative efficacy of lycopene in the cells exposed to smoke. These assays will be included in our future direction. Another limitation is that we failed to detect the protein expression of NTH1 by utilizing western blot due to the weak signaling. Although NTH1 mRNA expression showed positive correlation with its protein expression [55], we plan to reexamine its protein expression. It needs to be noted that we dissolved lycopene in DMSO, whereas ATRA and BC were dissolved in ethanol. This is because lycopene has extremely low solubility in ethanol, due to its high polarity (lycopene has more conjugated π bonds than BC) and large molecular size. Nevertheless, since the control groups were treated with the same solvents as the treatment groups at the same dosages, there was limited possibility that the lack of response of ATRA and BC was attributed to the different solvent. Finally, although we observed increased BER-enzyme protein levels, DNA repair may not be the only mechanism involving lycopene inhibition of smoke-induced oxidative stress. It is entirely possible that the final efficacy is the results of an equilibrium of different antioxidant mechanisms.

Taken together, we conclude that lycopene treatment at low-dosage can inhibit smoke-induced oxidative stress via upregulation of BER-related genes and carotenoid intake. Our current work supports the notion that low-dose lycopene supplementation can be employed as feasible means to prevent oxidative stress-induced lung cancer. However, further human research studies are needed to validate its functionality in clinical practice.

## Supplementary Materials

The following are available online at https://www.mdpi.com/2076-3921/9/7/643/s1, Table S1: Primers.

## Abbreviations

ATRA: all-trans retinoic acid, BC: β-carotene, OGG1: 8-oxoguanine DNA glycosylase, ROS: reactive oxygen species GJC: gap junction communication, 8-OHdG: 8-Hydroxy-2-deoxyguanosine, 8-oxoG: 8-oxoguanine BER:, Base excision repair, NTH1: Nth like DNA glycosylase 1, NEIL: Nei like DNA glycosylase, RIPA: radioimmunoprecipitation, CD36: cluster of differentiation, BCO1: beta-carotene oxygenase 1, BCO2: beta-carotene oxygenase 2, ANOVA: analysis of variance, SR-B1: Scavenger receptor type B class I.
