# Supplementary material for: Lycopene Protects against Smoking-Induced Lung Cancer by Inducing Base Excision Repair
Source: Antioxidants (Basel). 2020 Jul 21;9(7):643. doi: 10.3390/antiox9070643 (PMC7402151; doi:10.3390/antiox9070643)
Supplement: Supplementary file 1 [file antioxidants-09-00643-s001.pdf]

Supplementary Table 1

Gene	Forward Sequence	Reverse Sequence
β-Actin	CTTTTCCAGCCTTCCTTCTTGG	CAGCACTGTGTTGGCATAGAGG
SR-B1	GGCATTCCGATCAGTGCAACA	AGGGTTTTGGGAGCCACGAA
BCO1	GGCTTAGAGCTTCCACGGGT	GCCAGCAGTCGTCCTCTCTC
BCO2	TGCTGTAGGTAAGGGGAATGCT	GTGCAACACATGGCAGACCC
CD36	GCGACATGATTAATGGCACA	CCTGCAAATGTCAGAGGAAA
nth like DNA glycosylase 1 (NTH1)	AGCTGGTCAACATCCGTGCC	TGGCTGGAGAGCATCAGTGAC
